# Efficacy and safety of TCM therapies combined with hyperthermic intraperitoneal chemotherapy for peritoneal metastasis of gastric cancer: A protocol for systematic review and meta-analysis

**DOI:** 10.1097/MD.0000000000024337

**Published:** 2021-01-29

**Authors:** Xiaoyu Zhu, Zhe Wu, Yan Cao, Ruike Gao, Xiaoxiao Zhang, Jie Li

**Affiliations:** Oncology department, Guang ’anmen Hospital, China Academy of Chinese Medical Sciences, Beijing, China.

**Keywords:** complementary and alternative medicine, gastric cancer, hyperthermic intraperitoneal chemotherapy, meta-analysis, peritoneal metastasis, protocol, systematic review, Traditional Chinese Medicine

## Abstract

**Background:**

Gastric cancer (GC) has high incidence and mortality worldwide, and peritoneal metastasis is a primary cause of mortality in patients. Hyperthermic intraperitoneal chemotherapy (HIPEC) is a feasible and effective treatment. Traditional Chinese Medicine (TCM) therapies have been combined with HIPEC for certain therapeutic advantages, but there is a lacking of evidence of evidence-based medicine. Therefore, we provide a protocol to evaluate the efficacy and safety of TCM therapies combined with HIPEC in the treatment for peritoneal metastasis of GC.

**Methods and analysis:**

From inception until December 2020, a systematic and comprehensive literature search will be conducted in both 3 English databases and 4 Chinese databases. Randomized controlled trials (RCTs) will be included related to TCM therapies combined with HIPEC in the treatment for peritoneal metastasis of GC. Two researchers independently conducted data extraction and literature quality evaluation. The methodological qualities, including the risk of bias, will be evaluated using the Cochrane risk of bias assessment tool, while confidence in the cumulative evidence will be evaluated using the Grading of Recommendations Assessment, Development and Evaluation (GRADE) approach.

**Results:**

This study assessed the efficacy and safety of TCM therapies combined with HIPEC in the treatment of peritoneal metastasis of GC by effective rate, Karnofsky Performance Status (KPS), Carcinoemybryonic Angtigen remission rate, and incidence of adverse reactions etc.

**Conclusions:**

This study will provide reliable evidence-based evidence for the clinical application of TCM therapies combined with HIPEC in the treatment for peritoneal metastasis of GC.

**Ethics and dissemination:**

Ethical approval is not required, as this study is based on the review of published research. This review will be published in a peer-reviewed journal and disseminated both electronically and in print.

**Registration number:**

INPLASY2020120048.

## 1 Introduction

Gastric cancer (GC) representing 10% of all cancers is the fifth most common cancer worldwide, shifting from its spot as the most common malignancy in the last 40 years.^[1]^ The International Agency for Research on Cancer estimated that there were about one million new cases of gastric cancer and 782,685 cases of gastric cancer-related deaths in 2018, the third most common cause of death from cancer.^[2]^ Peritoneal metastasis is the most common metastasis of GC and considered incurable and alarming 50% of this mortality is associated with peritoneal metastasis even after the standard radical resection.^[3]^ Hyperthermic intraperitoneal chemotherapy (HIPEC) has been shown to be an effective treatment for peritoneal metastases from GC.^[4]^ However, compared with patients who received intravenous chemotherapy, patients who received HIPEC experienced higher toxicity and severer side effects (e.g., abdominal pain and bone marrow suppression) and complications (e.g., catheter obstruction and deteriorated renal functions). Thus, patients may be unable to complete their six-course conventional HIPEC.^[5]^

Traditional Chinese Medicine (TCM) therapies include TCM decoction and Chinese patent medicine. As complementary and alternative medicine, TCM has become one of main methods for cancer comprehensive treatment, because it has the advantages of improving the clinical symptoms, enhancing immunity and alleviating chemotherapy-induced adverse reactions.^[6,7]^ Although several studies have been conducted, the efficacy and safety of TCM therapies combined with HIPEC in the treatment of peritoneal metastasis in GC remain elusive.

Given the lack of a comprehensive evidence synthesis, we aim to assess the efficacy and safety of TCM therapies combined with HIPEC in the treatment of peritoneal metastasis in GC. Research on TCM is based on the Chinese Medicine New Medicine Clinical Practice Guideline (Trial Implementation) (published by China Medical Science Press in 2002) and TCM theory of combination of disease and syndrome.

## 2 Method

### 2.1 Study registration

**Figure 1:**
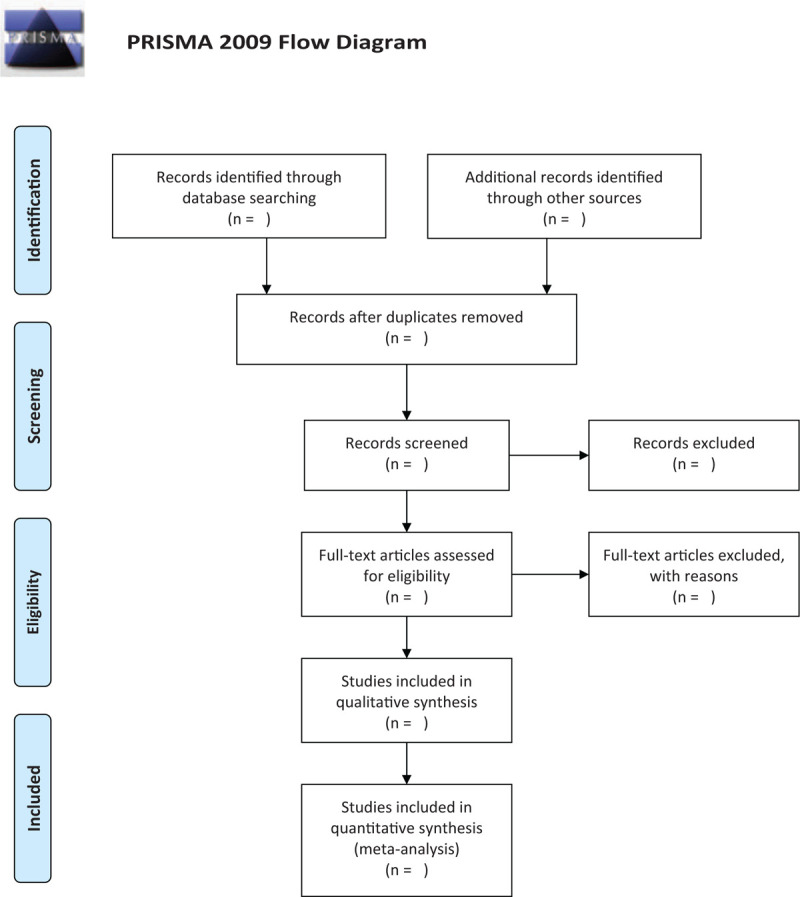
Flow diagram of studies search and selection.

This study will follow the guidelines outlined in the preferred reporting items for systematic reviews and meta-analysis (PRISMA) statement for meta-analyses of healthcare interventions;^[8]^ additionally, the protocol adheres to the PRISMA Protocols (PRISMA-P).^[9]^ The selection process will be summarized according to PRISMA flow diagram (Fig. 1).

### 2.2 Eligibility criteria

The eligibility criteria for the review using the PICOS (population-intervention-comparative-results-study design) framework are as follows.

#### 2.2.1 Population

Patients (adults over 18 years old) were histologically confirmed peritoneal metastasis of gastric cancer, and TNM classification was based on National Comprehensive Cancer Network.^[10]^

#### 2.2.2 Interventions/Comparators

Intervention: TCM therapy combined with HIPEC. TCM therapy interventions, including TCM decoction and Chinese patent medicine. Comparators: HIPEC alone.

#### 2.2.3 Outcome measures

Primary outcomes: disease-free survival (DFS); overall survival (OS).

Secondary outcomes: adverse effects; change in symptoms as measured with validated questionnaires; quality of life as measured using a validated questionnaire.

#### 2.2.4 Study design

Regardless of the blind method and language, only randomized controlled trials (RCTs) will be included. RCTs regarding TCM therapies plus HIPEC for peritoneal metastasis of gastric cancer.

### 2.3 Search strategy

The Cochrane Library, MEDLINE, Embase, Chinese BioMedical Database (CBM), China National Knowledge Infrastructure (CNKI), Chinese VIP Information (VIP), Wangfang Database will be searched regardless of publication date, or language. In order to obtain the potential nonelectronic literature, relevant magazines, and medical journals will also be filtered for further search. The search strategy of PubMed database is as follows (Table 1).

**Table 1:** Search strategy in PubMed database.

Number	Search terms
#1	Traditional Chinese Medicine [MeSH]
#2	Medicine, Chinese Traditional [Title/Abstract] OR Zhong Yi Xue [Title/Abstract]OR Traditional Medicine, Chinese [Title/Abstract] OR Chinese Herbal Drugs [Title/Abstract] OR Plant Extracts, Chinese [Title/Abstract] OR Chinese Plant Extracts [Title/Abstract] OR Chinese Herbal Drugs [Title/Abstract]
#3	#1 OR #2
#4	Gastric cancer [MeSH]
#5	Neoplasm, Stomach [Title/Abstract] OR Stomach Neoplasm [Title/Abstract] OR Neoplasms, Stomach [Title/Abstract] OR Gastric Cancer, Familial Diffuse [Title/Abstract] OR Gastric Neoplasm[Title/Abstract] OR Neoplasm, Gastric [Title/Abstract] OR Cancer of Stomach [Title/Abstract] OR Stomach Cancers [Title/Abstract] OR Cancer, Gastric [Title/Abstract]
#6	#4 OR #5
#7	Peritoneal metastasis [MeSH]
#8	Neoplasm, Peritoneal[Title/Abstract] OR Peritoneal Neoplasm[Title/Abstract] OR Peritoneal Surface Malignancy[Title/Abstract] OR Malignancy, Peritoneal Surface[Title/Abstract] OR Peritoneal Surface Malignancies [Title/Abstract] OR Surface Malignancy, Peritoneal[Title/Abstract] OR Peritoneal Carcinomatosis[Title/Abstract] OR Carcinomatosis, Peritoneal[Title/Abstract] OR Peritoneal Carcinomatoses[Title/Abstract]
#9	#7 OR #8
#10	Hyperthermic intraperitoneal chemotherapy[MeSH]
#11	Hyperthermic Intraperitoneal Chemotherapy[Title/Abstract] OR Chemotherapy, Hyperthermic Intraperitoneal [Title/Abstract] OR Hyperthermic Intraperitoneal Chemotherapies [Title/Abstract]
#12	#10 OR #11
#13	#3 AND #6 AND #9 AND #12

## 3 Data collection and analysis

### 3.1 Selection of studies

After removing duplicates, 2 reviewers (XZ and ZW) will independently evaluate the titles and abstracts of the searched studies by EndNote X9.0 (Stanford, Connecticut, https://endnote.com). Independently screen the included studies, extract data, evaluate the quality of included studies and cross-check with each other according to the established selection criteria. Any different opinions generated between the 2 reviewers will be resolved through discussion. When consultation fails to reach an agreement, the third reviewer (JL) will step in and provide arbitration. A PRISMA flow chart will be designed to illustrate the study selection procedure.

### 3.2 Data extraction and management

We will check the final results of the data extraction and extract the following data: last name of the author, publication time, study design, comparator, study period, numbers of outcomes, sex, age, locations, histologic diagnosis, BMI, Functional Assessment of Cancer Therapy (FACT) v4 score, and KPS score duration of TCM therapies, timing of TCM therapies, chemotherapy regimens, duration of follow-up, and relevant indicators of bias risk assessment. If above-mentioned information is not able to get, we will contact the corresponding authors for additional information.

### 3.3 Quality assessment

#### 3.3.1 Assessment of risk of bias

Two reviewers (XZ and ZW) will evaluate risk of bias in included studies based on the Cochrane collaboration's risk of bias assessment tool. If there are divided questions or opinions between 2 investigators will asking for a help from a senior researcher. Seven items are included: random sequence generation, allocation concealment, subjects and researchers blinded, outcome evaluation of blind method, the result data are incomplete and selective report results and other sources of bias and classified as “low,” “high,” or “unclear”.^[11]^

#### 3.3.2 Measures of treatment effect

We will apply relative risk (RR) to represent the enumeration data; measurement data will be represented by mean difference (MD) and 95% confidence interval (95% CI).

#### 3.3.3 Dealing with missing data

We will attempt to contact the author of the articles to obtain the missing data. We will explain the situation and use the available data to accomplish our analysis if it cannot be obtained.

#### 3.3.4 Assessment of quality in included studies

The quality of each selected studies will be evaluated using the Grading of Recommendations Assessment, Development and Evaluation (GRADE) approach by 3 investigators.^[12]^

#### 3.3.5 Assessment of heterogeneity

Statistical heterogeneity was statistically computed by using the chi-squared test and the inconsistency index statistic. If *I*^*2*^ ≤ 50% and *P* ≥ .05, it suggests that heterogeneity is not important and the Mantel-Haenszel fixed model will be employed for meta-analysis. If *I*^*2*^ > 50% and *P* < .05, it manifests that heterogeneity needs to be analyzed. If there is a high heterogeneity, we will conduct subgroup analyses to explore the possible causes.^[13]^

#### 3.3.6 Assessment of reporting bias

When more than 5 studies are included, visual dissymmetry on the funnel plot indicates whether there is publication bias. Reporting bias could be also assessed by using Egger test^[14]^ and Begger analysis.

### 3.4 Data synthesis

We will use RevMan 5.3 software (The Cochrane Collaboration, Oxford, England) to calculate for data synthesis. If there no obvious statistical heterogeneity among the trails included, we will apply fixed effects model to perform in the analysis. However, the random effects model will be used, when apparent clinical heterogeneity among the trails included. Meanwhile, subgroup or sensitivity analysis will be conducted. α = 0.05 will be deemed statistically significant.

### 3.5 Subgroup analysis

Subgroup analysis will be conducted according to sex, locations, histologic diagnosis, duration of TCM therapies, timing of TCM therapies, and chemotherapy regimens.

### 3.6 Sensitivity analysis

Sensitivity analysis will be conducted to explore the quality of studies of the document following sample size, the outcome of missing data, and methodological quality.

### 3.7 Ethics and dissemination

Ethical approval is not required because individual patient information will be not used. The authors will disseminate this systematic review through conference presentations and peer-review publications.

## 4 Discussion

Traditional Chinese medicine and other herbal medicines have been used to treat cancer in China, Korea, and Japan for more than thousands of years. Many studies were conducted to show TCM therapies combined with HIPEC associated with better DFS, OS, and quality of life.^[15–17]^ Taking these studies into consideration, TCM therapies could be recommended as a complementary method applied to GC patients undergoing peritoneal metastasis. But their overall efficacy and safety have not been evaluated scientifically and systematically in recent years. A systematic review which can provide the reliable data should be conducted to show such combination of therapeutic method associated with better DFS, OS, and quality of life. This systematic review aims to assess the effectiveness and safety of TCM therapies combined with HIPEC in the treatment of peritoneal metastasis of GC. We hope that our works will help clinicians with more convincing evidence.

## 5 Amendments

If amendments are needed, we will update our protocol to include any changes in the whole process of research.

## Author contributions

XZ (Xiaoyu Zhu) and ZW (Zhe Wu) are co-first authors of this manuscript, contributing equally to the design and drafting the manuscript. All authors participated in the design of the study and performed it. JL (Jie Li) is corresponding author of this manuscript. All authors read and approved the final manuscript.

**Conceptualization:** Xiaoyu Zhu, Zhe Wu, Yan Cao.

**Data curation:** Xiaoyu Zhu, Zhe Wu, Yan Cao.

**Investigation:** Xiaoyu Zhu, Zhe Wu, Ruike Gao, Xiaoxiao Zhang, Jie Li.

**Methodology:** Xiaoyu Zhu, Zhe Wu, Ruike Gao, Xiaoxiao Zhang.

**Project administration:** Jie Li.

**Resources:** Ruike Gao, Xiaoxiao Zhang.

**Software:** Ruike Gao.

**Supervision:** Xiaoxiao Zhang.

**Writing – original draft:** Xiaoyu Zhu, Zhe Wu.

**Writing – review & editing:** Ruike Gao, Xiaoxiao Zhang, Xiaoyu Zhu, Zhe Wu, Yan Cao, Jie Li.

## Abbreviations

Abbreviations: CI = confidence intervals, DFS = disease-free survival, GC = gastric cancer, HIPEC = Hyperthermic intraperitoneal chemotherapy, KPS = Karnofsky Performance Status, OS = overall survival, PRISMA-P = preferred reporting items for systematic reviews and meta-analyses protocols, RCT = randomized controlled trial, RR = relative risk, TCM = Traditional Chinese Medicine.
